# Jocks versus Geeks—the Downside of Genius?

**DOI:** 10.1371/journal.pbio.1001872

**Published:** 2014-05-27

**Authors:** Roland G. Roberts

**Affiliations:** Public Library of Science, Cambridge, United Kingdom

[Fig pbio-1001872-g001]Genomes seem to change relatively steadily through evolution, with an accumulation of mutations which have such a linear relationship to elapsed time that we can use them as a fairly reliable “molecular clock”. But because some mutations matter more than others and interact with each other in complex ways, the gross appearance and behaviour of species—the phenotype—can differ in quite spectacular ways even between closely related species. The genotype and phenotype are connected by layers of intermediate phenotype, including the characteristics of the transcriptomes, proteomes, and metabolomes of each tissue of an organism's body. It's by filtration through this information network that the consequences of genomic differences become manifest in the whole organism.

**Figure pbio-1001872-g001:**
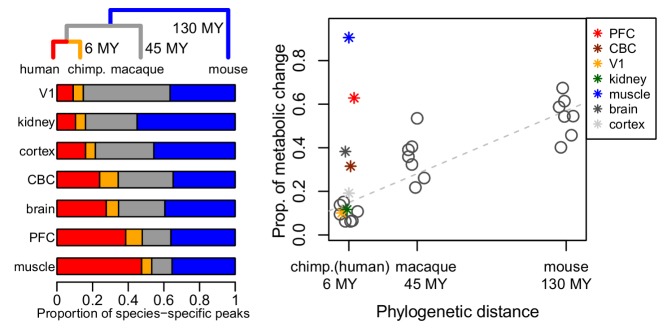
Phylogenetic distances and metabolome evolution. Not all tissues follow the molecular clock: while kidney and visual cortex (V1) accumulated metabolic changes proportionally to species' divergence time (MY = million years), metabolomes of skeletal muscle and the brain's prefrontal cortex (PFC) evolved exceptionally rapidly in the human lineage.

Somewhat narcissistically, one of the spectacular changes in phenotype that we tend to be most interested in is the enhancement in our own brain power which has occurred over the 6 million years that separate us from our last shared ancestor with chimpanzees. The chimp genome is famously very similar to our own, but the technological, linguistic, and cultural phenotype is clearly profoundly different. Several studies have asked open-ended questions as to what happens between the genotype and phenotype to make us so different from our cousins, finding differences in levels, splicing, and editing of gene transcripts, for example. Now a paper just published in *PLOS Biology* by Katarzyna Bozek, Philipp Khaitovich, and colleagues looks at another intermediate phenotype—the metabolome—with some intriguing and unexpected answers.

The metabolome is the set of small molecules (metabolites) that are found in a given tissue; by “small” we mean those with a molecular weight of less than 1,500 Daltons, which includes fats, amino acids, sugars, nucleotides, and vitamins (vitamin B12, for example, is near the top end of this range). These authors take five judiciously chosen tissues from 14 individual representatives of four judiciously chosen species and analyse 10,000 different metabolites in each one. Much of the rest of the study consists of detailed statistical scrutiny of the massive dataset that this matrix generates.

The selected tissues comprise prefrontal cortex (a part of the brain that we think is particularly special in humans), visual cortex (a more fitted-as-standard part of the cortex), cerebellum (an even more fundamental brain region), kidney, and muscle (two non-brain tissues). And in addition to humans, the species analysed are chimps, macaques, and mice, representing animals that split with our lineage 6, 45, and 130 million years ago, respectively.

After controlling for sex, age, and postmortem decay, the authors ended up with characteristic metabolomics profiles for each tissue and species. They were able to compare these with transcriptomic data to show that concentrations of metabolites tended to track the expression levels of associated enzymes in the various tissues. So far so good, but what they were really interested in were the differences between *species*.

Using the known genomic differences between the species as a yardstick to control for the expected degree of departure between animals with varying lengths of separate evolutionary history, the authors were able to home in on tissues that had changed unexpectedly quickly. On this basis, they show that the metabolomes of human prefrontal cortex (and of combined brain regions) have changed four times as rapidly in the last 6 million years as those of chimps. While gratifying, this largely confirms for metabolites what was already known for transcripts.

What is much more unexpected, however, is that brain is not the most spectacular outlier here. The real surprise is that the human *muscle* metabolome has experienced more than eight times as much change as its chimp counterpart. Indeed, metabolomically speaking, human muscle has changed more in the last 6 million years than mouse muscle has since we parted company from mice back in the Early Cretaceous. Most of the changes relate to pathways involved in the metabolism of carbohydrates and amino acids, and in energy production. By contrast, the visual cortex and kidney faithfully track the rate of genomic change.

What's the significance of this striking metabolomic remodelling of muscle during human evolution? The authors ran a short-term experiment whereby macaques were subjected to aspects of the modern human “couch potato” lifestyle for 2 months (limited exercise, stress, fatty and sugary diet), but this explained only 3% of the changes. They also rule out changes in muscle fibre type composition–another obvious explanation. They wondered then whether these recent metabolic changes in the human muscle might instead reflect a unique physical phenotype.

The literature on the comparative muscle performance of humans and other primates is very limited, with a few anecdotal suggestions that humans might be weaker. To address this, the authors compared the performance of humans, chimps, and macaques in a strength test that involved pulling a handle to raise a weight. Human strength, as measured by this test, was barely half that of the non-human primates. Amazingly, untrained chimps and macaques raised in captivity easily outperformed university-level basketball players and professional mountain climbers.

The authors do point out that the strength test has some severe limitations which complicate a straight comparison. For example, the species tested have different distributions of muscle between arms and legs (both of which are used in the task), and the degree of motivation might vary (the non-humans were motivated by a food reward). In addition, the geometrical relationship between the different species and the apparatus might affect the leverage available, and the animals might opt to use different muscle groups. So this experiment should probably be seen as a tantalising preliminary enquiry as to what the evident metabolomic differences in muscle might mean.

The take-home message of this paper is therefore that, even when many variables are taken into account, human brain and muscle have experienced rapid and pronounced species-specific changes in metabolite profiles since we split from our closest extant relatives, the chimps. The physiological implications of this remain unclear—the authors provide suggestive evidence for a negative impact on strength, but there might also be consequences for other aspects of energy management, such as those involved in endurance—and further experiments are obviously needed to resolve this. The authors speculate that the fates of human brain and muscle may be inextricably entwined, and that weak muscle may be the price we pay for the metabolic demands of our amazing cognitive powers.

**Bozek K, Wei Y, Yan Z, Liu X, Xiong J, et al. (2014) Exceptional Evolutionary Divergence of Human Muscle and Brain Metabolomes Parallels Human Cognitive and Physical Uniqueness.** doi:10.1371/journal.pbio.1001871

